# What to Do in Cases of Poisoning

**Published:** 1887-09

**Authors:** 


WHAT TO DO IN CASES OF POISONING. By William Murrell, M. D., F. R. C. P. First American, from Fifth English Edition. Medical Register Co., Philadelphia. 1887. Pp. 158. Price $1.00.

A book which should be on every practitioner's table for ready reference. It is practical, concise and definite, with directions which it would benefit every medical man to read and keep handy for use when called to a case of poisoning. With such sources of knowledge accessible, there is no excuse for ignorance or incapacity. H. H.

